# Plasma-assisted three-dimensional lightscribe graphene as high-performance supercapacitors

**DOI:** 10.1038/s41598-022-08315-9

**Published:** 2022-03-11

**Authors:** Naser Namdar, Foad Ghasemi, Zeinab Sanaee

**Affiliations:** 1 Nano-Fabricated Energy Devices Laboratory, School of Electrical and Computer Engineering, College of Engineering, University of Tehran, Tehran, Iran; 2 Nanoscale Physics Device Lab (NPDL), Department of Physics, University of Kurdistan, Sanandaj, Iran

**Keywords:** Electronic properties and devices, Electrochemistry

## Abstract

Graphene-based supercapacitors demonstrate extraordinary energy storage capacity due to their layered structure, large effective surface area, high electrical conductivity and acceptable chemical stability. Herein, reduced graphene oxide (rGO)-based supercapacitors were introduced in a simple, and fast method with considerable performance. For this purpose, graphene oxide (GO) was synthesized by the modified Hummers’ method and then easily reduced to desired patterns of rGO using a commercial LightScribe DVD drive. In order to increase the effective surface area, as well as the electrical conductivity of rGO layers, oxygen/sulfur hexafluoride plasma was applied to the rGO followed by laser irradiation. By performing such sequential processes, an rGO-based supercapacitor was introduced with a capacitance of about 10.2 F/cm^3^, which had high stability for more than 1000 consecutive charge–discharge cycles. The fabrication steps of the electrodes were investigated by different analyses such as SEM, TEM, Raman, surface resistance, BET, and XPS measurements. Results showed that these rGO-based electrodes fabricated by sequential processes are very interesting for practical applications of energy storage.

## Introduction

Tremendous demands of energy conversion and storage attracted scientists and researchers in recent years to investigate solutions to address this need^1^. Novelty in the fabrication of rechargeable batteries, fuel cells (FC) and supercapacitors which are the typical electrochemical energy storage devices requires important strategies to fulfill the emerging markets, particularly transportation applications and portable smart electronic^2^. In this regard, supercapacitor plays an important role due to their ability to short charging time, high power density and long cycling life^3^. Carbon derivatives are considered as a plausible candidate for supercapacitor electrode active material and have been studied extensively thanks to their long-term stability, crystalline structure and high electrical conductivity^4^. Among the carbon family, graphene has attracted more attention because of its large surface area, promising flexibility, tensile strength and ease of functionalization^5^. Graphene is a one-atom-thick planar sheet consisting of sp^2^ carbon atoms that are densely packed in a honeycomb crystal lattice^6^. In the last decade, graphene derivatives such as graphene oxide (GO) have received more attention than intrinsic graphene due to easy synthesis and hydrophilicity^7^. However, from a conduction point of view, GO is an insulator and shows a poor charge transport characteristic for energy applications^8^. Hence, several methods have been introduced to improve the electrical properties of GO which leads to the reduced graphene oxide (rGO), including thermal, chemical, UV-assisted, and light scribed reduction techniques^9^. Thermal treatment is time-consuming, costly, unsafe and requires high operating temperatures for effective reductions^8–10^. Chemical reductions are not only toxic but also lead to the agglomeration of graphene sheets^11^. In addition, they usually form nitrogen bonds in graphene, which strongly affect its electrical properties^12^. Although UV-assisted is a green method, it is nevertheless less efficient than other methods in reducing GO^8^. Compared to these methods, lightscribe reduction method has a significant advantage due to its green, easy, fast and effective reduction of GO^13^. In this method, the GO solution is cast either directly onto a DVD or a flexible layer and then exposed to laser light inside the DVD burner. The laser wavelength of 780 nm helps to break the oxygen bonds and reduces the GO layers. The width of the laser beam is about 20 µm and it can reduce GO to the designed pattern of rGO layers line by line. This process can be done quickly and in a few minutes. Finally, the rGO film is removed from the surface of the DVD to employ in an LSG based electronic device. Farther, efforts carried out to improve the efficiency of obtained graphene by altering physical and electrical properties of flakes by means of doping, compositing and functionalization, made it one of the key topics in graphene research recently^14^. The chemical functionalization of graphene is increasingly considered as a novel method to tune its physical, chemical and electrical properties^15^. This process can be categorized into two major approaches: direct anchoring of dopant atoms onto basal plane^16^ and establishing covalent bonds between functional groups of GO and guest atoms^17^. In this regard, chemical functionalization can be obtained by doping graphene flakes with halogen elements such as nitrogen (N), boron (B), sulfur (S) and fluorine (F) atoms, turning it from a gapless to a gapped structure of either n- or p-types depending on the dopant types^18–20^. In fact, highly electronegative halogen atoms could bind to carbon stronger than hydrogen to form a much more stable complex^21^. Among these elements, fluorine is the most electronegative element and as a result, the C–F bond is expected to demonstrate high polarity nature and excellent oxidative and thermal stability^22,23^. Furthermore, fluorinated graphene (FG) is at the center of extensive research in the field of electronics, optics, as well as energy storage applications due to the open new possibilities of structural engineering^24^. Up to now, several methods have been employed to prepare FGs such as high-temperature exposure to fluorine gas or room temperature xenon difluoride (XeF_2_) irradiation^25,26^. As an alternative approach, FGs could be prepared by a sulfur hexafluoride (SF_6_) plasma-assisted process instead of F_2_ and XeF_2_ sources which are also less toxic and more compatible with IC fabrication processes^27^. Another advantage of this method is that the fluorination process is accompanied by partial etching of graphene flakes, which leads to three-dimensional FGs^28^. This 3D structure can dramatically increase electrochemical active sites and surface area of obtained FGs which are excellent for supercapacitor applications. Hwee Ling Poh et al. introduced a microwave plasma exfoliation of graphite oxides in SF_6_, SF_4_ or MoF_6_ containing atmospheres to obtain FGs^29^. In addition to the effective doping of graphene, they improved the electrochemical properties of the flakes. Resultant doped graphene sheets also take advantage of more defect sites introduced during the doping process making them suitable for electrochemical applications. Moreover, the presence of fluorine bonds in graphene leads to interesting electrochemical properties such as fast heterogeneous electron transfer rates [A]. Akshay Mathkar et al. demonstrated that the low surface energy of the C–F bond changes GO’s wetting properties, leading to amphiphobicity in its highly fluorinated form^30^. Although most of these introduced methods result in an increase of the surface area of graphene, they generally associate with a decrease in conductivity, which greatly reduces the charge transfer rate at the surface of the electrode for electrochemical applications. Therefore, more effective methods are needed to overcome this issue.

In this work, a controllable method is employed to introduce 3D fluorinated LSG electrodes that show superior performance compared with other reports. First, GO flakes are synthesized by modified Hummer’s method and the reduction process is performed with the help of a commercial ready lightscribe computer optical drive laser diode. In a precise and controlled process, the impact of laser radiation-assisted reduction and oxygen and fluorine plasma bombardments are investigated on prepared layers. GO is electrically insulating, and the laser reduction process turns it into conductive layers of rGO, but still provides a small surface area. In order to further improve the conductivity and effective surface area of the rGO layers, laser reduction processes and SF_6_ + O_2_ plasma bombardment are combined to finally obtain the best-case scenario as a suitable electrode for supercapacitor application. Plasma irradiation not only leads to the formation of a 3D rGo structure but also the fluorine bonds remaining in the layer by locally altering the electronic structure of rGo play an important role in improving electrochemical properties. The electrochemical testing of all introduced electrodes indicated that lightscribe reduction of the 15 min SF_6_ + O_2_ plasma-treated rGO sample shows the highest capacitance of ~ 10.2 F/cm^3^ compared with other approaches due to its larger surface area and higher conductivity. Finally, the practical performance of the introduced rGO based supercapacitor was tested by lighting up a red LED showing very high promising potential in energy-storing applications.

## Results and discussion

**Figure 1:**
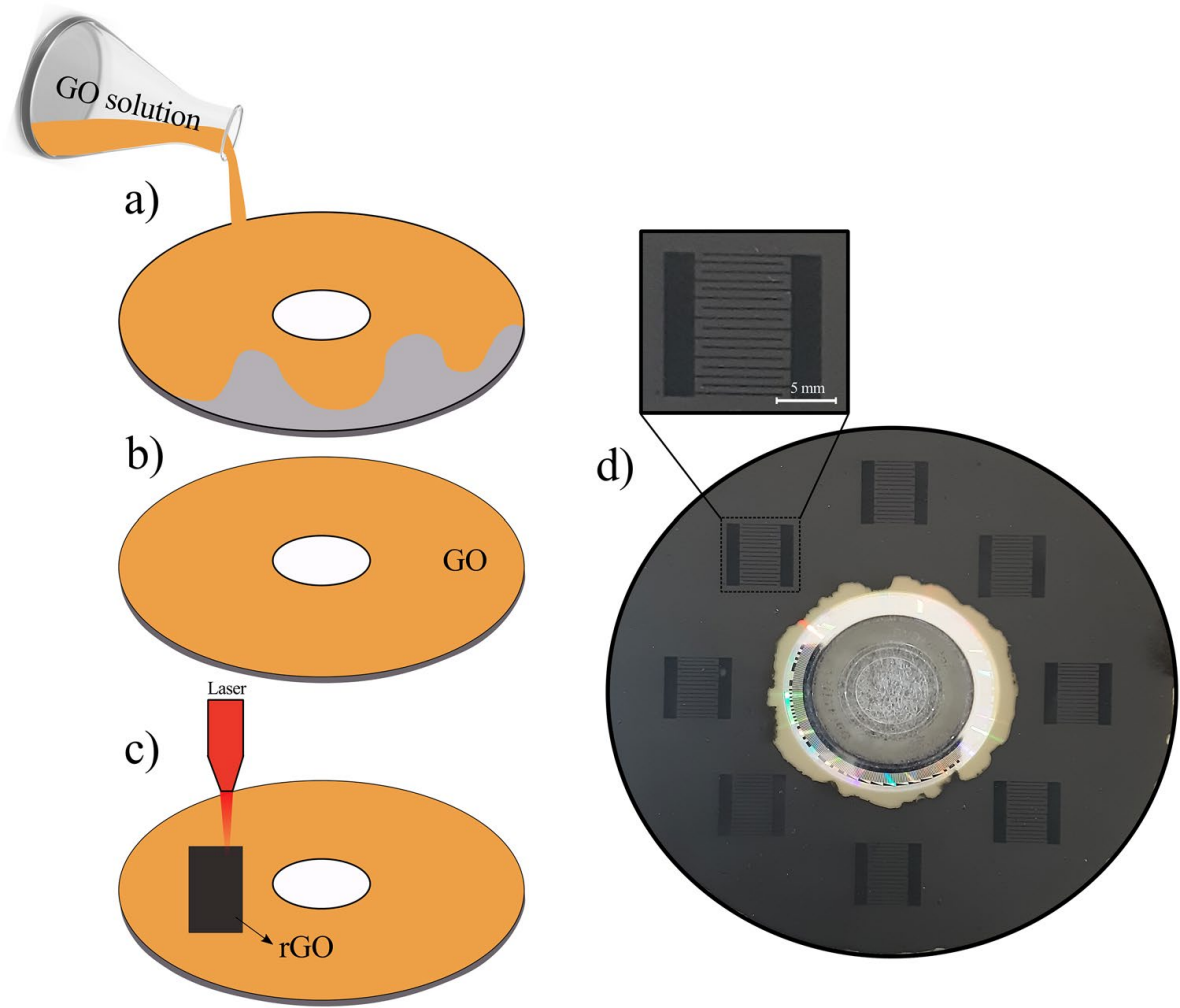
Schematic of (**a**) GO solution casting on a DVD, (**b**) dried GO film, and (**c**) lightscribe GO called rGO, (**d**) Photograph of the patterned rGO on the DVD.

The GO solution was synthesized by the modified Hummers method, the synthesis details are described in the “Experimental” section^31^. The prepared solution was cast onto a commercial DVD and allowed to dry completely. The GO-coated DVD is then inserted into the lightscribe driver. The commercial DVD burner has a 780 nm laser with a diameter of 20 μm, which scans the surface of a GO-containing DVD to convert it to rGO. By designing the path, laser scanning can reduce GO into any desire rGO patterns. This reduction process can be done in few minutes and rGO can prepare quickly in a controlled manner. Figure 1a–c shows the schematic of casting GO solution on a commercial DVD and reduction process by the lightscribe driver. The photograph of the lightscribe patterned rGO on the DVD is shown in Fig. 1d, in which the dark patterned layers refer to the rGO and the lighter parts are the GO film.

**Figure 2:**
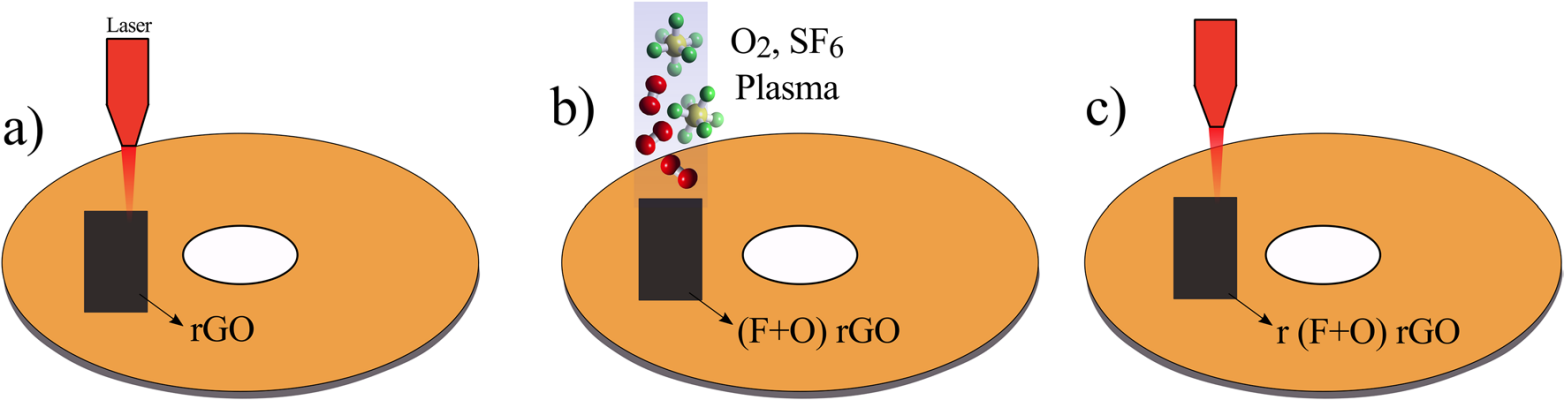
(**a**) Reduction of GO into rGO by the lightscribe method. (**b**) Plasma treatment of prepared rGO with SF_6_ and O_2_ gases. (**c**) Lightscribe reducing of the (F + O)rGO sample.

In order to increase the effective surface area of rGO layers for supercapacitor applications, the prepared films were exposed to oxygen (O_2_) and sulfur hexafluoride (SF_6_) gases at room temperature. Accordingly, various controlled processes of plasma irradiations and lightscribe reduction were integrated to get the best results. In this regard, the following samples were prepared: GO, rGO, SF_6_ plasma-treated GO named as FGO, SF_6_ + O_2_ plasma-treated GO labeled as (F + O)GO, lightscribe FGO named as rFGO, lightscribe (F + O)GO called r(F + O)GO, SF_6_ plasma-treated rGO named as FrGO, lightscribe FrGO called rFrGO, and finally, lightscribe (F + O)rGO labeled as r(F + O)rGO. The schematic of the r(F + O)rGO fabrication process is presented in Fig. 2. Based on this, the GO solution is first cast on a DVD and after drying, it converts into rGO by the lightscribe method (Fig. 2a). The reduced part is then exposed to SF_6_ and O_2_ containing plasma for 5 min (Fig. 2b). This process led to the formation of a three-dimensional structure of rGO on the substrate. However, functional groups originating from ion bombardments can enter into the rGO layers and degrade their reduction rate^32^. The sample is then re-irradiated with the laser to facilitate the elimination of the functional groups introduced during plasma bombardment (Fig. 2c). In fact, these sequential processes contribute to the formation of a three-dimensional, high conductive and large surface area rGO structure. In fact, by evaluating the laser reduction and plasma steps, we showed that the best electrode for energy storage can be introduced.

**Figure 3:**
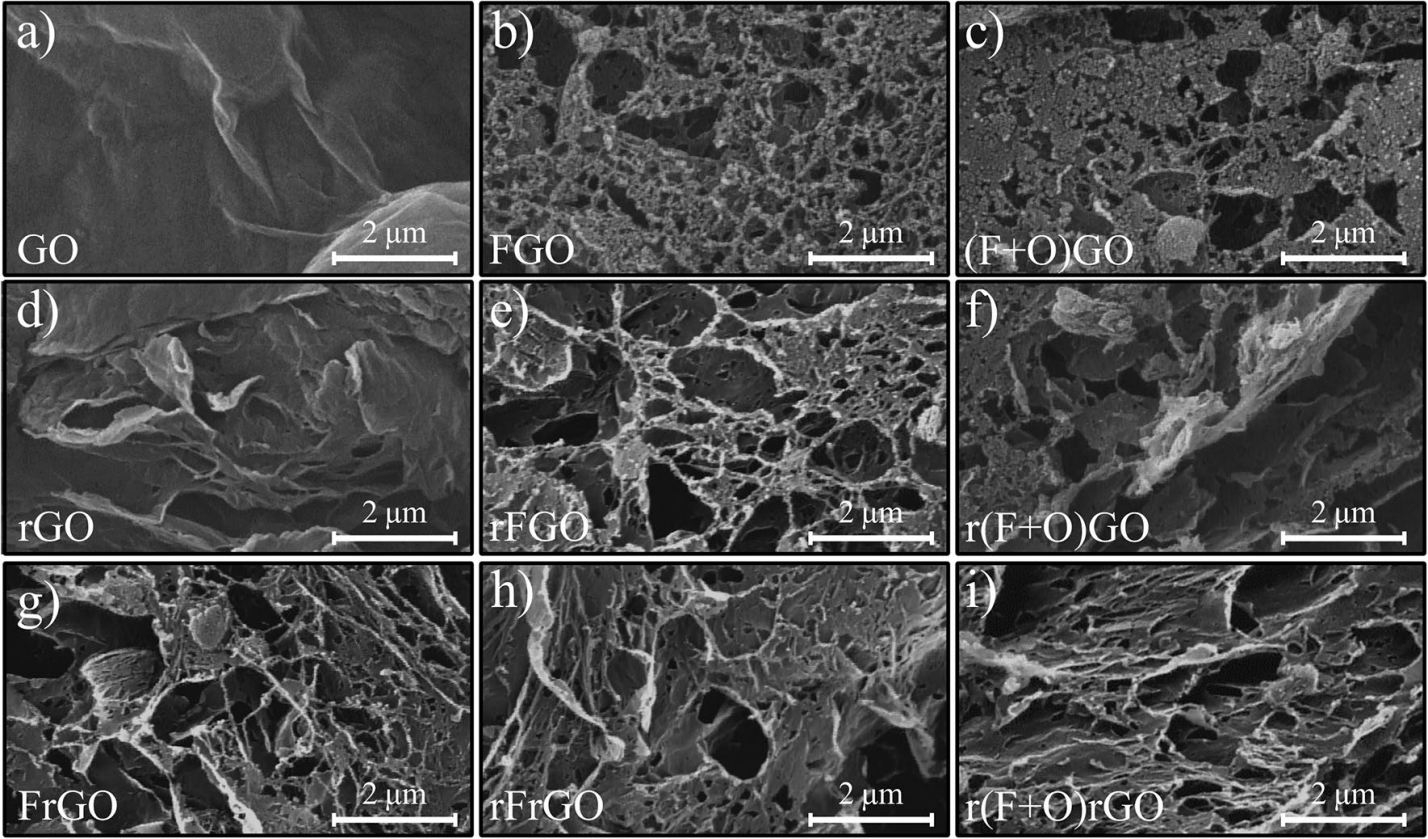
FE-SEM image of the (**a**) pristine GO, (**b**) FGO, (**c**) (F + O)GO, (**d**) rGO, (**e**) rFGO, (**f**) r(F + G)GO, (**g**) FrGO, (**h**) rFrGO, and (**i**) r(F + O)rGO samples.

Figure 3 shows the SEM images of all prepared samples. The morphology of the GO layers is seen in Fig. 3a. After SF_6_ plasma treatment, it is observed that most of these layers are etched as shown in Fig. 3b. In the case of (F + O)GO, the addition of O_2_ gas leads to a decrease in the etching rate of SF_6_ and deeper penetration of the fluorine radicals which results in the controlled formation of porosity, as can be seen in Fig. 3c. After lightscribe reduction of GO, the layered structure of rGO sheets can be seen due to the expansion of layers as a result of the gasification of oxygen functional groups^33^ (Fig. 3d). A similar trend is observed for rFGO in comparison with FGO as shown in Fig. 3e. By lightscribe irradiation of (F + O)GO, it expects to be as the r(F + O)GO. According to Fig. 3f, the expanded layers originate from the reduction process which shows that the underlying sheets are less exposed to plasma and subsequently have less porosity. Comparison of FGO and FrGO samples also demonstrates that in the latter there are more opened layers with higher porosity, which highlights that the plasma treatment affects more effectively on the rGO than GO in creating porosity (Fig. 3g). The rFrGO sample also displays that the second reduction process can remove some of the heavily etched layers from the film due to further expansion and gasification of fluorine functional groups, as can be seen in Fig. 3h. Generally, the expanded rGO sheets can effectively interact with applying plasma ions compared with GO sheets. Moreover, the SEM images of Sf_6_ and SF_6_ + O_2_ plasma-treated samples show that the latter results in a much greater porosity and larger surface area. Therefore, according to SEM results, it can be concluded that the r(F + O)rGO sample offers a more three-dimensional structure in comparison to other introduced samples (Fig. 3i).

**Figure 4:**
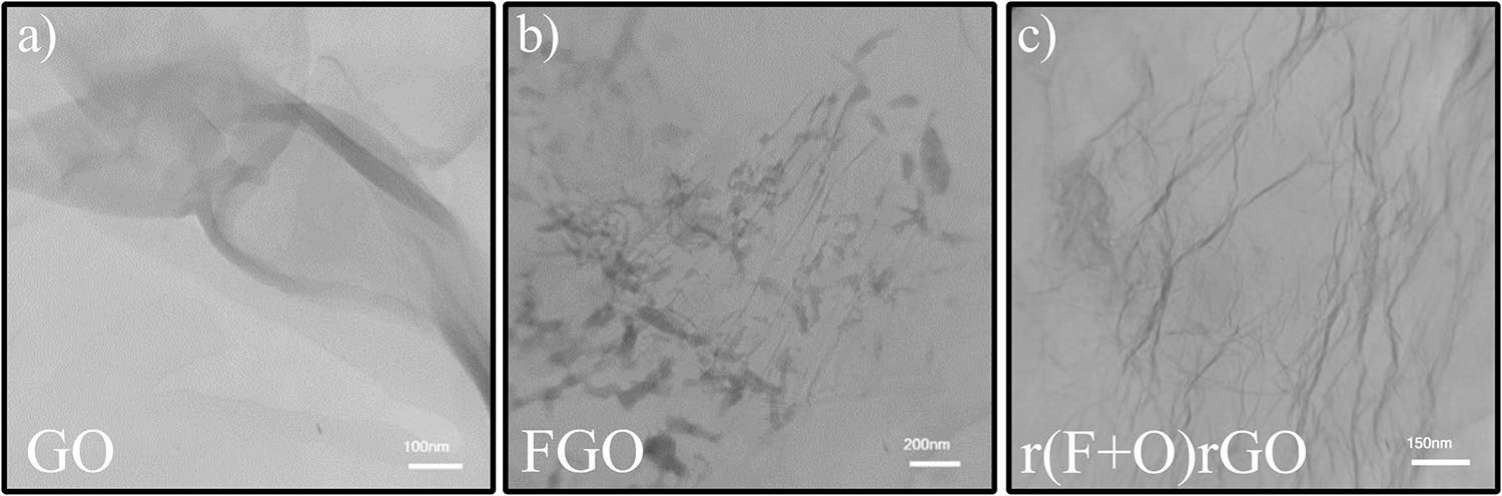
TEM image of the (**a**) pristine GO layer, and (**b**) FGO layer, and (**c**) r(F + O)rGO layer.

Figure 4a shows the TEM image of GO sheets synthesized by the modified Hummers method where are exfoliated by the sonication process. The layered structure of the GO sheets is well visible in the image. TEM image of the one FGO layer is presented in Fig. 4b. Accordingly, FGO is locally etched after SF_6_ plasma treatment and lost its integrated layered form. Moreover, the TEM image of the r(F + O)rGO sample exhibits its layered structure with more wrinkles due to reduction and plasma treatment processes (Fig. 4c).

**Figure 5:**
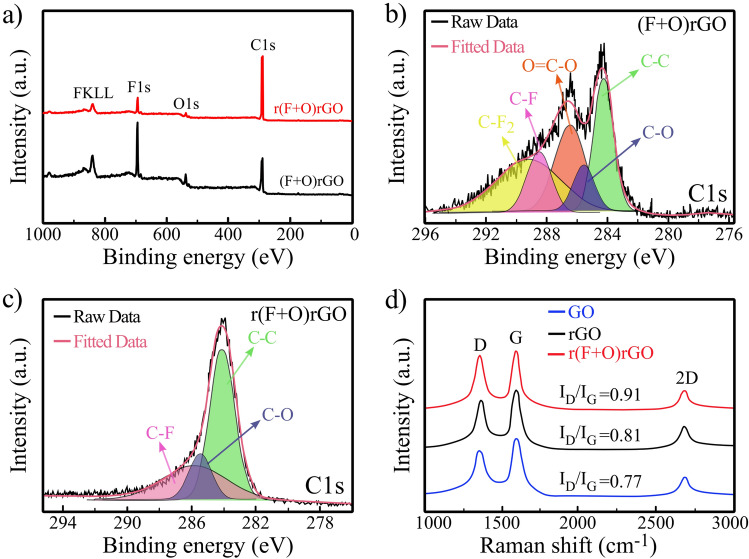
(**a**) XPS survey spectra of (F + O)rGO, and r(F + O)rGO samples. (**b**) The C1s XPS spectrum of the (F + O)rGO sample. (**c**) The C1s XPS spectrum of the r(F + O)rGO sample. (**d**) Raman spectra of the GO and rGO and r(F + O)rGO samples.

Figure 5a compares the XPS survey spectra of r(F + O)rGO and (F + O)rGO samples. Four peaks of C1s, O1s, F1s and FKLL are observed in both spectra. In the case of r(F + O)rGO sample, the intensity of O1s and fluorine peaks show a decrease relative to the intensity of C1s peak that refers to an effective role of the lightscribe step in eliminating these functional groups. Figure 5b presents the C1s XPS spectrum of the (F + O)rGO sample. Accordingly, strong C-F and C-F_2_ bonds appear after O_2_ + SF_6_ plasma treatment that refers to the effective fluorination of rGO sheets^34^. Vacancy defects in the rGO are reasons for introducing these fluorine bonds in the plasma-exposed rGO^35^. Although the formation of fluorine bonds improves the energy storage capacity of the treated rGO layers, it greatly reduces their conductivity, which will be discussed later. Therefore, the second reduction step is necessary to improve the conductivity of the (F + O)rGO sample. Figure 5c refers to the C1s XPS spectrum of the r(F + O)rGO sample in which the contents of fluorine and oxygen elements decrease thanks to the lightscribe process that supplies the energy needed to eliminate these functional groups. It is also expected that the conductivity of the r(F + O)rGO significantly increases. As can be seen in Fig. 5c, after the second reduction process, a small content of fluorine and oxygen bonds remain, which can locally change the electronic structure of rGO (due to the high electronegativity of the fluorine atoms) and play an effective role in increasing the specific capacity of the r(F + O)rGO^36^. In fact, in this work, the combination of oxygen + fluorine plasma has been used for two purposes. Fluorine alone etches a large proportion of rGo, which drastically reduces the electrical conductivity while adding oxygen slows down the etch process because the product of its reaction with fluorine gas is highly volatile SOF_4_ that reduces the density of fluorine radicals^37^. In addition, XPS results show that fluoride ions remain within rGO even after the lightscribe step to some extent, which promotes the electrochemical properties of the rGO^38^. Fluorine bonds not only improve the electrochemical reactions via generating polar states, but also increase the dielectric constant of GO, which leads to an increase in energy storage capacity^38^.

The Raman spectra of the GO, lightscribe GO (rGO), and r(F + O)rGO are shown in Fig. 5d. As can be seen, all samples exhibit typical disorder D, graphitic G, and amorphous 2D oscillation modes at around 1351, 1585, and 2590/cm, respectively^39^. The I_D_/I_G_ ratio is calculated for all three samples, which can show a degree of defects or change of the sp^2^ domain in the graphene layers^8,40,41^. According to the results, for GO, the value of this ratio is 0.77, and increases to 0.81 for rGO, which may be due to the increase in the number of smaller sp^2^ domains in rGO^41,42^. Interestingly, in the r(F + O)rGO sample, this ratio increases to 0.91 arising from the formation of defects in layers due to the bombardment and removal of some F-containing bonds after the second laser scribe reduction step^43^.

**Figure 6:**
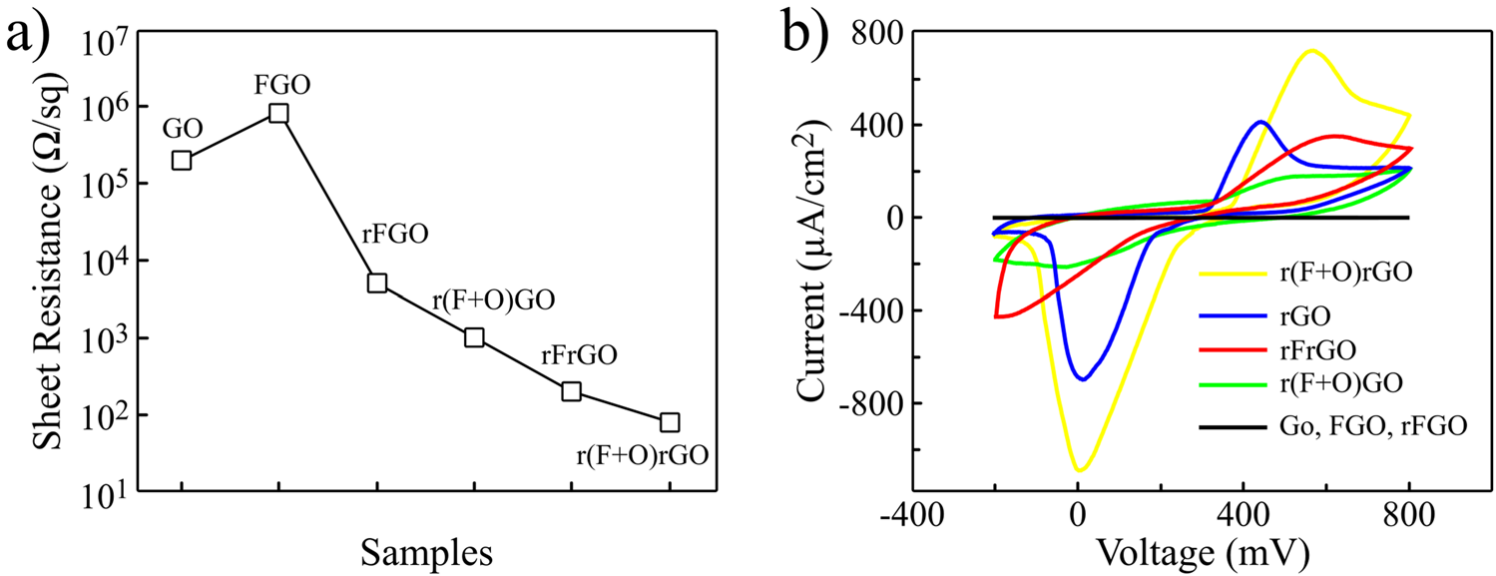
(**a**) Sheet resistance of Go, FGO, rFGO, r(F + O)GO, rFrGO, r(F + O)rGO samples. (**b**) Cyclic voltammetry tests of GO, FGO, rFGO, r(F + O)GO, rFrGO, r(F + O)rGO in a 5 mM K_3_Fe(CN)_6_ + 100 mM KCl aqueous solution.

Figure 6a displays the surface resistance measurement of all prepared samples. Based on it, the r(F + O)rGO sample has a resistance of about 82.8 Ω/sq that is the lowest surface resistance among all samples. Its comparison with rFrGO sample shows that the use of combined O_2_ and SF_6_ plasma gases results in a more efficient reduction of plasma-treated rGO. The reason behind this fact is due to the impact of oxygen ions that promote the penetration of fluorine radicals into deeper layers and provide more interaction with rGO. As a result, the second lightscribe process reduces the plasma-treated rGO layers more effectively. After SF_6_ bombardment of GO sheets, the resistance of the FGO sample increases, which can be due to the replacement of oxygen-related functional groups with fluorine, as well as the local etching of layers. After lightscribe irradiation, the resistance decreases in rFGO due to the elimination of surface fluorine and deeper oxygen functional groups. Interestingly, the electrical resistance of r(F + O)GO is lower than that of rFGO, indicating the effective role of oxygen ions in its improvement. Furthermore, by applying plasma to the rGO samples lower resistance can be achieved compared with GO samples because the reduction process leads to the opening of the layers and provides larger surfaces for interaction with plasma ions. The electrochemical tests of all fabricated electrodes are also presented in Fig. 6b. All samples were tested in a 5 mM K_3_Fe(CN)_6_ with 100 mM KCl supporting electrolyte at a scan rate of 50 mV/s to evaluate their electrochemical performance as a reliable and standard method. To perform these tests, all samples were cut into 1 × 1 cm^2^ pieces and used in a three-electrode standard electrochemical cell as working electrodes where platinum and Ag/AgCl were used as a counter, and reference electrodes, respectively. Due to the high reduction capacity of Fe^3+^ ions, reduction peaks are observable in cyclic voltammetry tests. Comparison of these peaks is a very quantitative parameter to compare the electrochemical activity of the working electrode. According to the results, The r(F + O)rGO sample has the highest electrochemical activity. By comparing the reduction peaks of the samples, it can be seen that the highest current of 748 μA is measured for r(F + O)rGO followed by 412 μA for rGO. Therefore, r(F + O)rGO electrode demonstrates better electrochemical performance compared to other rGO samples. Interestingly, in the rFrGO sample, the reduction peak occurs at 373 μA, which has a degraded performance than rGO. The oxygen-free plasma means limits the porosity to the surface of graphene where not allow the fluorine radicals to penetrate the deeper layers. Therefore, the incorporation of reduction and SF_6_ + O_2_ plasma treatments which is result in r(F + O)rGO electrode offers more porosity, higher conductivity and larger surface area that deliver superior electrochemical performance.

**Figure 7:**
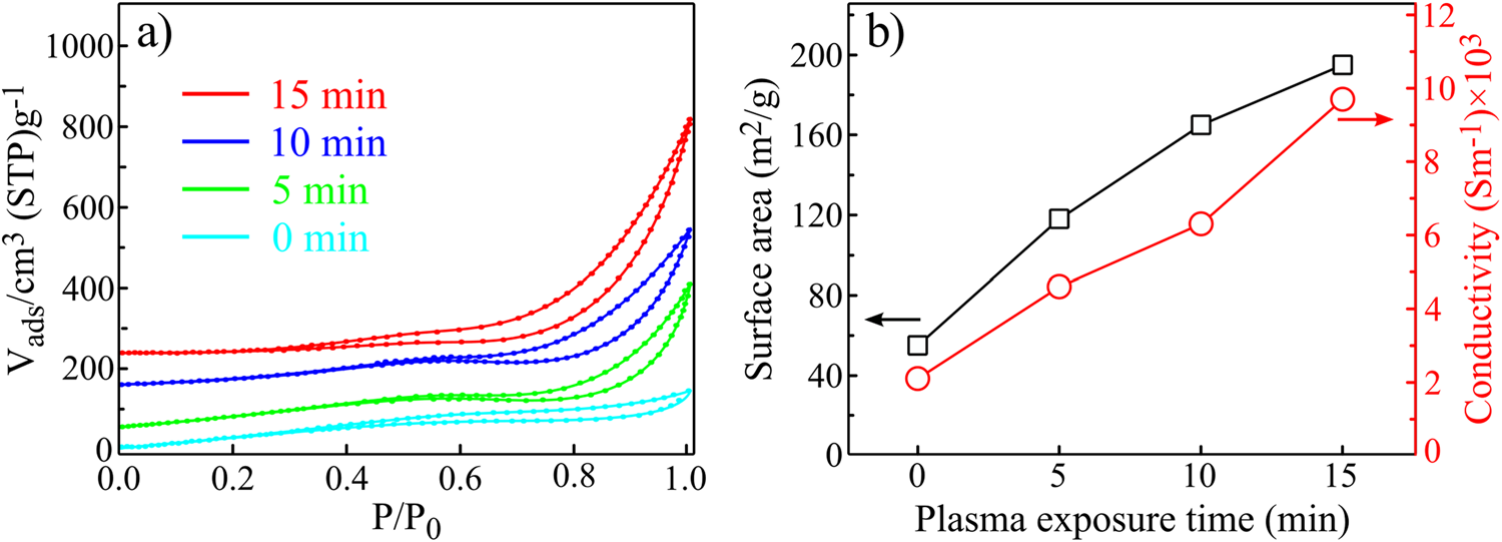
(**a**) N_2_ adsorption–desorption isotherms, (**b**) BET surface area and conductivity of r(F + O)rGO samples with different SF_6_ + O_2_ plasma exposure times.

Figure 7a shows the nitrogen isotherms on r(F + O)rGO with different plasma exposure times, which proves that 15 min treated sample provides larger specific areas than the other. Hysteresis is also observed at 0.4–1 relative pressure range, which indicates the presence of meso and slit-shaped pores caused by plasma irradiation^44^. The calculated surface area and the conductivity of the samples are also presented in Fig. 7b. Accordingly, by increasing the plasma time from 0 to 15 min, the effective surface areas are increased for the layers. In detail, the surface areas of 55, 118, 165 and 195 m^2^/g were measured for 0, 5, 10 and 15 min treated samples, respectively. The interesting point is that the conductivity of the samples also improved with the same trend, so that for 0 to 15 min plasma treatments, it increases from 2100 to 9700/Sm, respectively.

**Figure 8:**
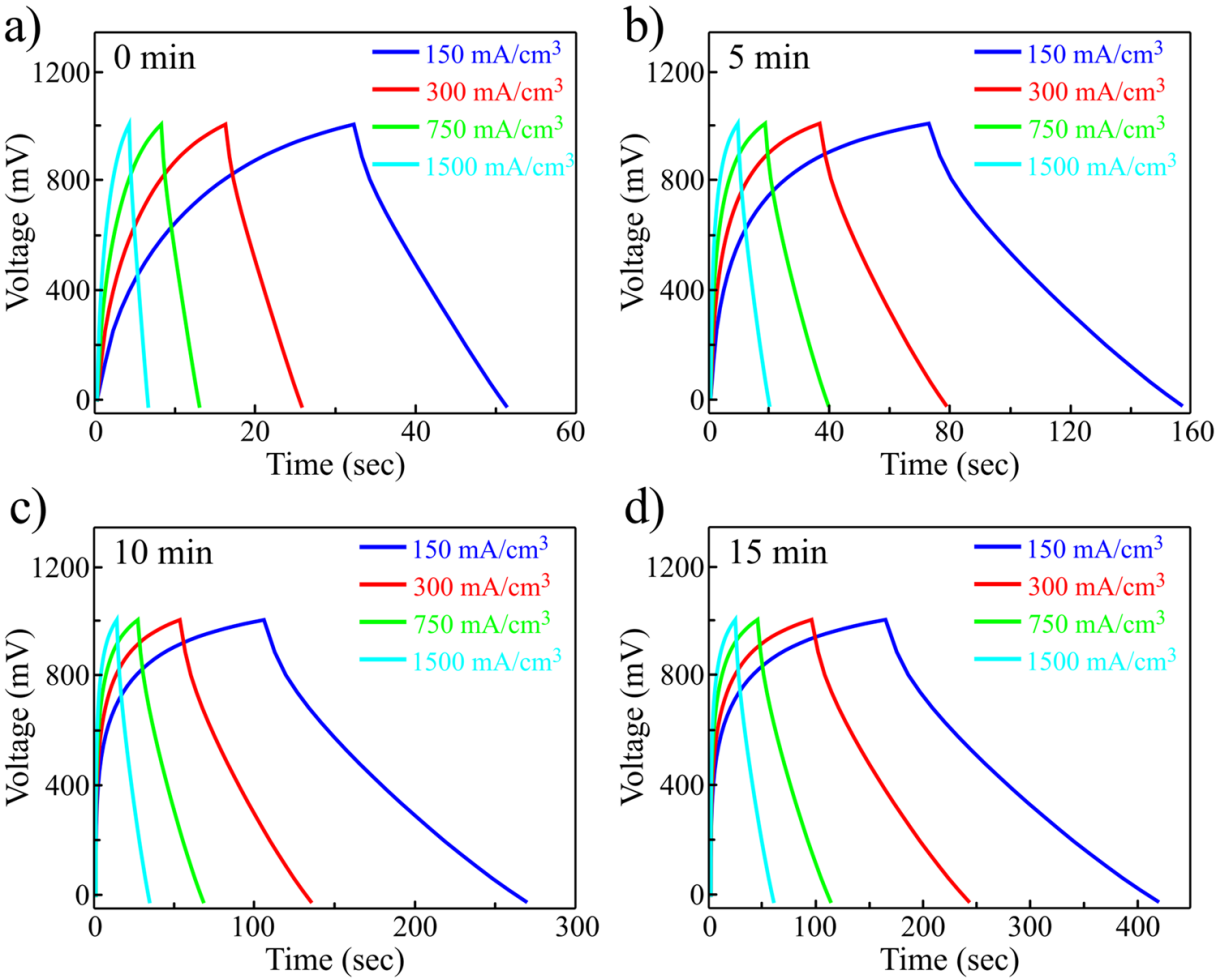
Charge–discharge curves of r(F + O)rGO supercapacitors with SF_6_ + O_2_ plasma exposure times of (**a**) 0 min, (**b**) 5 min, (**c**) 10 min, and (**d**) 15 min tested in PVA + H_2_SO_4_ electrolyte.

After determining the electrode with the highest amount of electrochemical activity based on the Fig. 6b, the charge and discharge curves of the r(F + O)rGO based supercapacitors with different plasma exposure times from 0 to 15 min were investigated in a three-electrode electrochemical cell using hydrogel electrolyte composed of PVA + H_2_SO_4_. The charging-discharging currents have been normalized per effective volume of rGO in the electrodes (see “Supporting information S1”). According to Fig. 8a–d, by increasing the plasma exposure times, charging and discharging times have increased significantly. Furthermore, the coulombic efficiency parameter which is equal to the ratio of discharge time to charging time is calculated for 0-, 5-, 10- and 15-min plasma-exposed samples equivalent to 72%, 89% and 94%, and 96%, respectively. Moreover, prolonged plasma exposure time leads to higher supercacitors performance where the capacitance can be accurately calculated using the slope of the discharge cycle according to follow:

1 $$ {\text{C}}_{{{\text{device}}}} = {\text{I}}/\left( { - {\text{dV}}/{\text{dt}}} \right) $$

The normalized capacitances of the 0-, 5-, 10-, and 15-min plasma-exposed electrodes were calculated around 1.1, 5.2, 8.0, and 10.2 F/cm^3^ for the first discharge cycle, respectively (see “Supporting information” and Figs. S1 and S2). The obtained results show that r(F + O)rGO with 15-min plasma exposure time terminates in the highest final performance of the supercapacitors as it improves the capacitance by up to 10 times compared to the case without plasma treatment. This result is also consistent with the measured surface areas and conductivities that offers the superiority of the 15-min plasma treatment sample compared with other samples. Table 1 shows the calculated volumetric and gravimetric capacitances of the 0–15 min plasma-exposed samples for samples at current densities of 150 mA/cm^3^ and 1 A/g, respectively. It is noteworthy that with increasing the plasma exposure time from 15 to 30 min, the effective thickness of the rGO sample decreases, and thus the capacitance decreases significantly. The Fig. S3 shows the changes in capacitance per unit volume as a function of F + O plasma bombardment time. It is observed that the maximum capacitance is obtained for the plasma exposure time of 15 min while further increase of plasma exposure time is accompanied by a significant decrease in specific capacitances.

**Table 1:** Gravimetric and volumetric capacitances of the 0–15 min plasma-exposed supercapacitors.

Plasma exposer time (min)	Capacitance (F/g) (At 1 A/g)	Capacitance (F/cm^3^) (At 150 mA/cm^3^)
0	16.14	1.1
5	76.32	5.2
10	117.42	8.0
15	149.72	10.2

**Figure 9:**
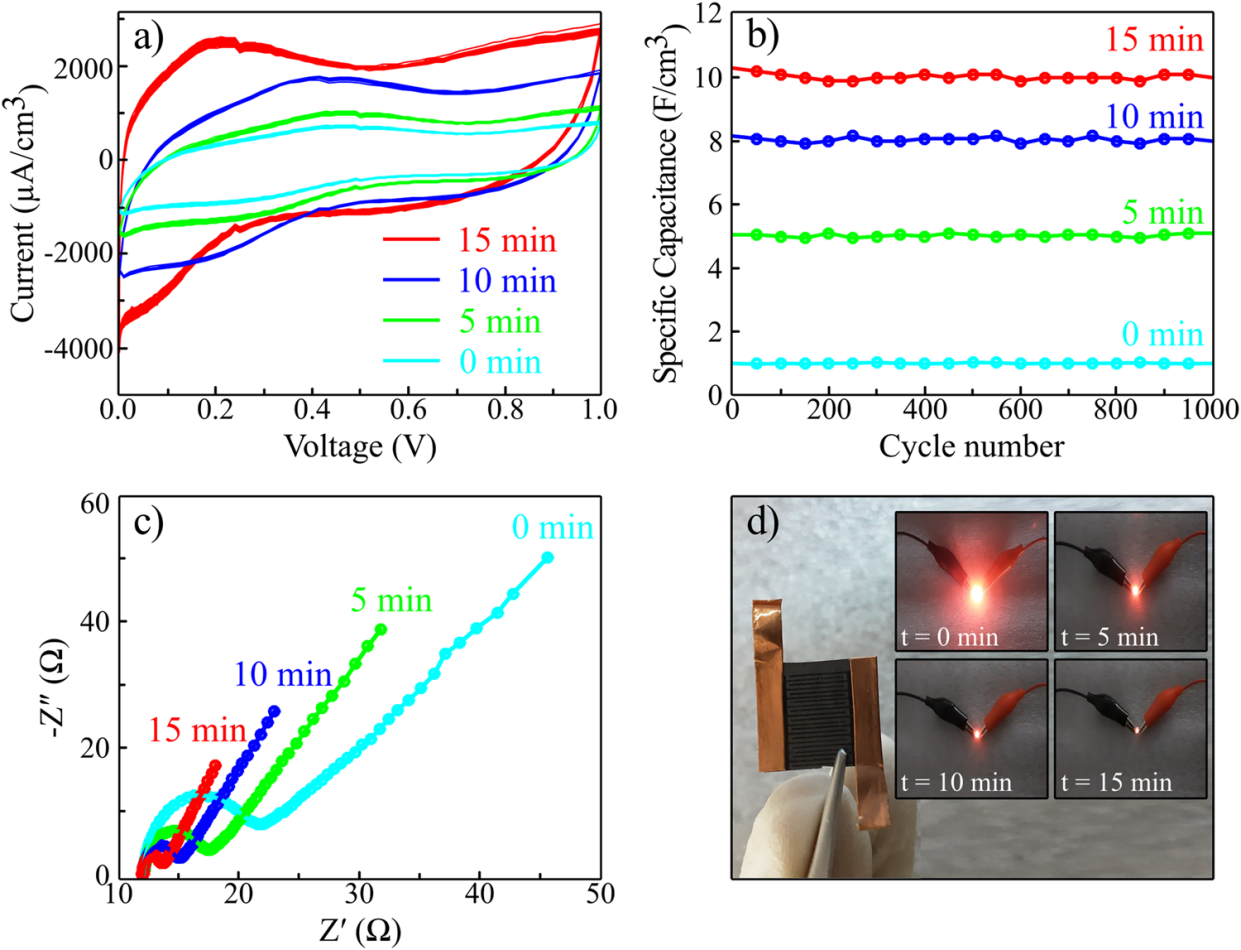
(**a**) Cyclic voltammetry test of r(F + O)rGO samples with different plasma exposure times from 0 to 15 min for 50 selected cycles from 1000 to 1050. (**b**) Stability test of resulted capacitances for 1000 continuous charge–discharge cycles. (**c**) Electrochemical Impedance Spectroscopy of the obtained electrodes with different plasma treated times. (**d**) Photograph of the two-electrode 15 min plasma treated r(F + O)rGO supercapacitor. Insets: photograph of the red LED being lighted up by the corresponding supercapacitor.

After determining the efficiency of r(F + O)rGO sample in the case of electrochemical activity and capacitance, we proceed to fabricate and test an rGO flat supercapacitor using H_2_SO_4_ + PVA polymer gel. The cyclic voltammetry test of the r(F + O)rGO supercapacitor with different plasma exposure times is presented for 50 consecutive cycles in Fig. 9a. The overlapping of the CV curves for each sample refers to their stable performance. To test the supercacitors, one terminal of the supercapacitor is connected to both counter and reference ports at the same time, and the other terminal is connected to the working port of the potentiostat. The area inside the cyclic voltammeter curve shows the amount of power density stored by the supercapacitor. As can be seen, the internal area of the 15 min plasma exposed r(F + O)rGO electrode (red curve) has the highest value compared to other samples that mean higher power and high capacitance. Figure 9b shows the capacitance of the r(F + O)rGO supercapacitors with different plasma exposure times for 1000 consecutive cycles. Accordingly, the capacitance remains constant as the charge and discharge cycles increase which indicates the high-stable performance of these supercapacitors. Moreover, r(F + O)rGO electrode with 15 min plasma treatment demonstrates an average capacitance of 10 F/cm^3^, higher than all reported supercapacitors based on lightscribe technique. Figure 9c presents the results of EIS analysis of supercapacitors upon different plasma exposure times. With increasing plasma irradiation time, EIS curves have shifted towards smaller real and imaginary values, referring to an increase in the relative conductivity of electrodes and efficient charge exchanges. Moreover, the slope of the linear part of the curves has increased as plasma time is increased, which demonstrates a larger Warburg capacitance. The 15 min plasma-treated r(F + O)rGO electrode has a larger surface area (according to the BET results) due to its higher porosity compared with other electrodes which give a higher chance of EDL (Electrical double layer) formation in the vicinity of the electrode. From the point of view of equivalent series resistance (ESR), the EIS curves show no significant change in the resistance of electrodes with different exposure times. Since the operating voltage range of aqueous hydrogel polymer electrolyte-based devices cannot exceed 1 V, that make them unusable for some applications. Therefore, using this device in a tandem structure can adapt them for higher voltage applications. Figure 9d presents the 15 min-r(F + O)rGO based two-electrode supercapacitor that is charged with a polymer gel electrolyte in a charging cycle of 0.95 V. Inset of Fig. 9d also shows the practical performance of two r(F + O)rGO micro super capacitors in series with a charging cycle of 1.9 V to illuminate a light-emitting diode (LED) with an average current of 820 µA, keeping it on for 23 min.

Table 2 shows the performance of lightscribe graphene-based supercapacitors and their comparison with our introduced supercapacitors. Accordingly, the specific capacitance obtained in our work is about 10.2 F/cm^3^, which shows a significant improvement compared to the capacitance of other works reported in the range of 134.0 mF/cm^3^–3.0 F/cm^3^.

**Table 2:** Performance comparison of different lightscribe graphene-based supercapacitors.

Electrode material	Specific capacitance	Refs.
r(F + O)rGO	10.2 F/cm^3^	This work
Lightscribe graphene	2.3 F/cm^3^	^45^
Lightscribe graphene	2.9 F/cm^3^	^46^
Lightscribe graphene	0.1 F/cm^3^	^47^
rGO	17.9 mF/cm^3^	^48^
Fluorinated graphene	134.0 mF/cm^3^	^49^

## Conclusion

The low-cost production, high conductivity and acceptable chemical stability of graphene make it suitable for supercapacitor applications. Herein, a new method is introduced based on lightscribe reduction and plasma exposure of graphene oxide to fabricate highly conductive three-dimensional reduced graphene oxide (rGO) films with a large surface area that offer capacitances of ~ 10 F/cm^3^ with a remarkably stable cycling life. Our results showed that 15 min bombardment of rGO sample with O_2_ and SF_6_ gases terminated in the best supercapacitor performance. O_2_ and SF_6_ plasmas create more porosity on the rGO surface than SF_6_ plasma, and laser lightscribe reduction helps to improve the conductivity of the plasma-treated rGO and reduces the fluorine functional groups. SEM, Raman, BET, XPS and surface resistance measurements were used to control the electrode fabrication process. In general, such a supercapacitor shows high potential in charge exchange reactions with high stability, which is a promising candidate for energy storage applications.

## Methods

### Graphene oxide synthesis

GO was prepared by the modified Hummers’ method as reported elsewhere. Briefly, 2 g graphite powders were added to a mixture of 1 g NaNO_3_ and 46 ml H_2_SO_4_ and the mixture was cooled to 10 °C using an ice bath. In the next step, 6 g KMnO_4_ was gradually added to the above solution while the reaction temperature was maintained below 20 °C. The mixture was then stirred at 35 °C for 2 h and diluted by adding 92 ml of distilled water until a dark brown suspension was obtained. Then, the suspension was treated by adding 340 ml H_2_O_2_ solution. The resulting graphite oxide suspension was washed several times by 10% HCl aqueous solution and distilled water. Finally, a uniform suspension of GO nanosheets was obtained by dispersing the resulting precipitate in distilled water and sonicated for 12 h. The resulting suspension was uniformly cast on the laser-scribing DVD disk and then dried under air ambient.

### Plasma treated process

The samples were exposed to SF_6_ and SF_6_ + O_2_ plasmas generated in a reactive ion etcher (RIE) system which operates at a radio-frequency (RF) of 13.56 MHz. The base pressure of the RIE system was 50 mtorr and an SF_6_ flow of 150 sccm was introduced into the chamber for 2 min. In the case of SF_6_ + O_2_ plasma, both gases fed into the chamber at the same flow rate of 100 sccm. An RF power of 150 W was also used in all experiments at room temperature.

### Lightscribe GO preparation

The pristine and plasma-treated GO coated DVDs were placed in a LightScribe DVD drive with a wavelength of 780 nm and a laser spot size of 20 μm. The reduced film was easily separated from the DVD disk and glued to PET substrates.

### Electrochemical tests

The GO and rGO samples were removed from the surface of the DVDs and glued to the PET substrates with the dimension of 1 × 1 cm^2^. The prepared electrodes were wired out by copper wire using silver paste and the exposed areas of silver paste were passivated by epoxy glue. The electrochemical testing of supercapacitors was evaluated in a three-electrode system with prepared samples as working electrodes, platinum rod as the counter electrode, and a standard Ag/AgCl as the reference electrode in 1 M H_2_SO_4_ solution as electrolyte. The gel electrolyte for supercapacitor testing was composed of H_2_SO_4_ and PVA polymer. Shortly, 6 g PVA was added to 6 g H_2_SO_4_ and dissolved into 60 ml DI water followed by stirring at 85 °C to become a clear solution. A proper amount of gel electrolyte was cast on the sample and then dried at room temperature.

### Characterizations

FE-SEM images of the prepared electrodes were captured by S-4160 Hitachi at 20 kV operating voltage. TEM images of the samples were taken by CM-30, Philips microscope at 150 kV operating voltage. Raman spectra of GO and rGO were measured by Senterra unit under 532 nm wavelength laser source. X-ray photoelectron spectroscopy (XPS) test was performed by Bestec instrument with Al Kα source at 1.4 keV. Sheet resistances were recorded by the Signatone Four-point probe system. Cyclic voltammetry (CV) and galvanostatic charge–discharge tests were carried out on a potentio/galvanostat CompactStat ivium system. The Electrochemical Impedance Spectroscopy (EIS) measurements were performed in the frequency range from 0.1 Hz to 100 kHz with CompactStat ivium system.

## Supplementary Information

Supplementary Information.
