# Supplementary material for: Plasma-assisted three-dimensional lightscribe graphene as high-performance supercapacitors
Source: Sci Rep. 2022 Mar 11;12:4254. doi: 10.1038/s41598-022-08315-9 (PMC8917148; doi:10.1038/s41598-022-08315-9)
Supplement: Supplementary file 1 — Supplementary Information. [file 41598_2022_8315_MOESM1_ESM.docx]

Supporting Information

**Plasma-assisted Three-Dimensional Lightscribe Graphene as High-Performance Supercapacitors**

Naser Namdar^1^, Foad Ghasemi^2^*, Zeinab Sanaee^1^*

^1^Nano-fabricated Energy Devices Laboratory, School of Electrical and Computer Engineering, College of Engineering, University of Tehran, Tehran, Iran.

^2^Nanoscale Physics Device Lab (NPDL), Department of Physics, University of Kurdistan, Sanandaj, Iran.

* Corresponding authors: [F.Ghasemi@uok.ac.ir](mailto:F.Ghasemi@uok.ac.ir), [Z.Sanaee@ut.ac.ir](mailto:Z.Sanaee@ut.ac.ir)

**Capacitance calculation**

The process of calculating the specific capacitance of the fabricated supercapacitors as well as normalizing the capacitance per unit area and effective volume is discussed here:


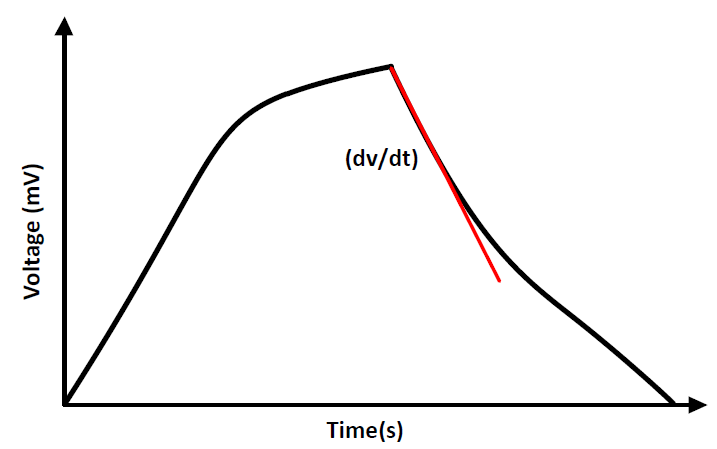


**Figure S1**. Effective slope of the discharge curve to calculate the capacitance.

The capacity of the supercacitors is calculated mainly through charge and discharge curves. For this purpose, the supercapacitor should be tested for significant consecutive cycles (1000 cycles) of charge/discharge to ensure the reproducibility and stability of the output data which thus increase the accuracy of performed calculations.

An important parameter in calculating the capacitance is the rate of voltage drop per unit time during the supercapacitor discharge process. It should be noted that voltage changes in the discharge area have mainly exponential behavior, which in order to avoid the entry of nonlinear parameters, the initial slope of the discharge area is taken into account in the calculations. Therefore, the tangent line is plotted on the discharge region according to the figure S1 and the total capacitance is calculated in terms of Farad according to follow:

C _Supercapacitor_ = i/(-dV/dt) (S1)

Where i is the charge and discharge current of the supercacitor.

To normalize the calculated capacitance per unit area and volume, the above values are divided by the effective surface (A) and the effective volume (V) according to the following formulas:

C_Areal_ = C_Supercapacitor_ /A (S2)

C_Volumetric_ = C_Supercapacitor_ /V (3)

In the case of planar supercapacitors, the effective area of ​​the supercapacitor (A) includes the area of ​​each of the positive and negative electrodes that act as the current collector plus the area of ​​the separator between the electrodes. This area is independent of the supercapacitor packaging. According to our calculations, the area of the ​​flat supercapacitors is equal to 92.34 mm^2^. To calculate the effective volume (V), the amount of effective surface must be multiplied by the thickness of the LSG, which acts as the active material. According to the cross-sectional SEM image of the samples, the effective thickness of the LSG is about 32 μm. Therefore, the effective volume of rGO flat supercapacitors is calculated to be 2.955 mm^3^, and all data, including current and capacitance, are normalized based on this value.


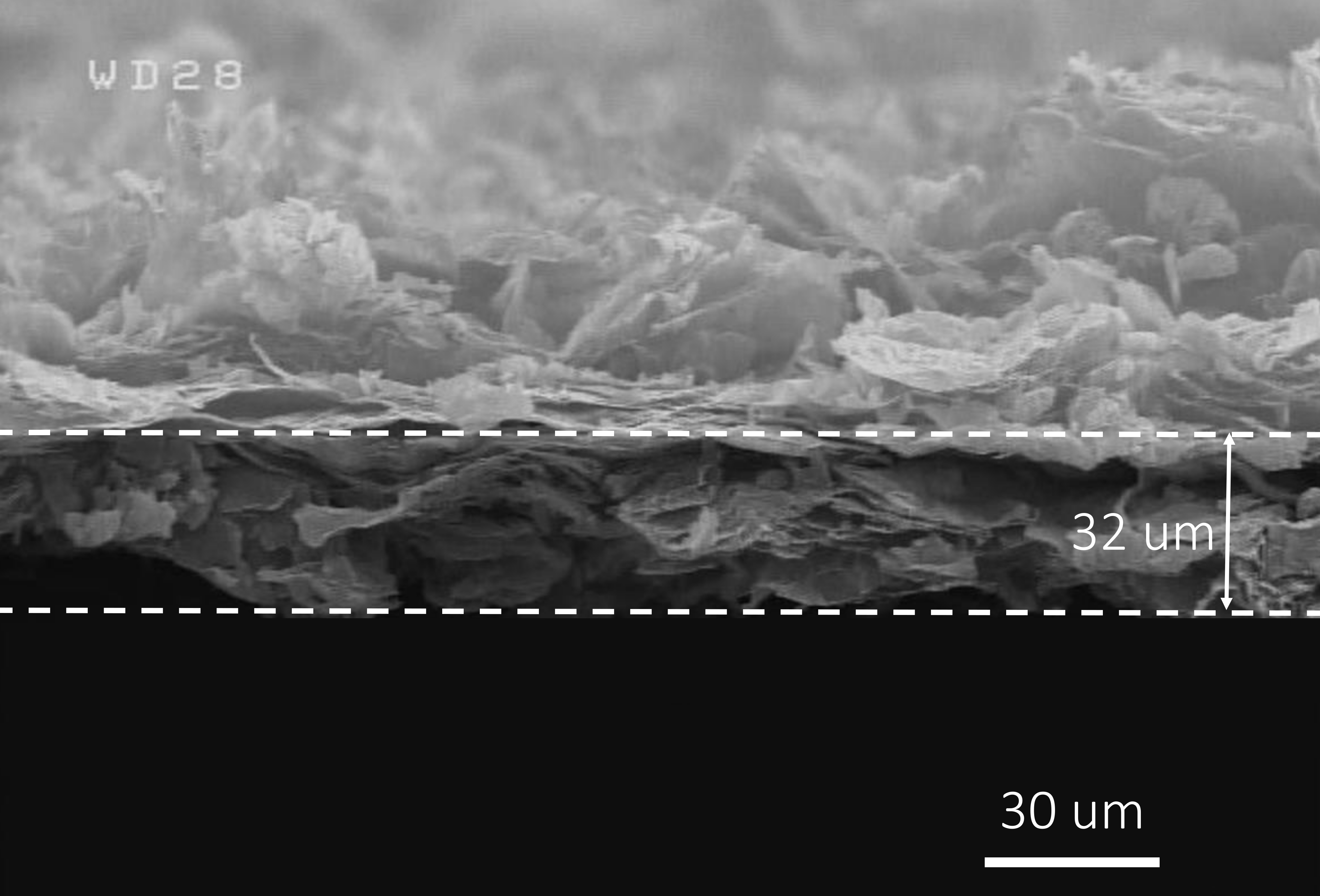


**Figure S2.** Cross-sectional image of the rGO film.


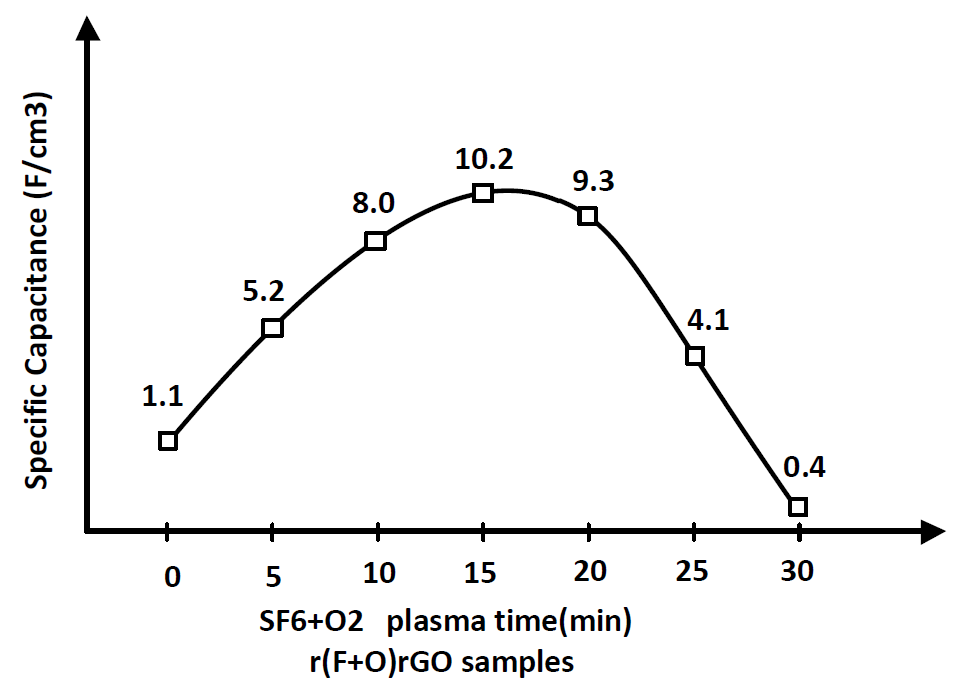


**Figure S3**. Specific capacitance per unit volume versus plasma exposure time increasing from 0 to 30 min.
